# *Mycobacterium tuberculosis* is less likely to acquire pathogenic mutations during latent infection than during active disease

**DOI:** 10.1128/spectrum.04289-23

**Published:** 2024-05-24

**Authors:** Asami Osugi, Aki Tamaru, Takashi Yoshiyama, Tomotada Iwamoto, Satoshi Mitarai, Yoshiro Murase

**Affiliations:** 1 Department of Mycobacterium Reference and Research, Research Institute of Tuberculosis, Japan Anti-Tuberculosis Association, Kiyose, Tokyo, Japan; 2 Department of Infectious Diseases, Osaka Prefectural Institute of Public Health, Osaka, Japan; 3 Research Institute of Tuberculosis, Japan Anti-Tuberculosis Association, Kiyose, Tokyo, Japan; 4 Department of Respiratory Medicine, Fukujuji Hospital, Japan Anti-Tuberculosis Association, Kiyose, Tokyo, Japan; 5 Kobe Institute of Health, Kobe, Hyogo, Japan; 6 Basic Mycobacteriology, Nagasaki University Graduate School of Biomedical Sciences, Nagasaki, Japan; University Paris-Saclay, Clamart, France

**Keywords:** latent infection, *Mycobacterium tuberculosis*, adaptive mutations

## Abstract

Most people infected with *Mycobacterium tuberculosis* (*Mtb*) are believed to be in a state of latent tuberculosis (TB) infection (LTBI). Although LTBI is asymptomatic and not infectious, there is a risk of developing active disease even decades after infection. Here, to characterize mutations acquired during LTBI, we collected and analyzed *Mtb* genomes from seven Japanese patient pairs, each pair consisting of two active TB patients whose starting dates of developing active disease were >3 years apart; one had a high suspicion of LTBI before developing active disease, whereas the other did not. Thereafter, we compared these genomes with those of longitudinal sample pairs within a host of chronic active TB infections combined with public data. The bacterial populations in patients with LTBI were genetically more homogeneous and accumulated single nucleotide polymorphisms (SNPs) slower than those from active disease. Moreover, the lower proportion of nonsynonymous SNPs indicated weaker selective pressures during LTBI than active disease. Finally, the different mutation spectrums indicated different mutators between LTBI and active disease. These results suggest that the likelihood of the acquisition of mutations responsible for antibiotic resistance and increased virulence was lower in the *Mtb* population from LTBI than active disease.

**IMPORTANCE:**

Controlling latent tuberculosis (TB) infection (LTBI) activation is an effective strategy for TB elimination, where understanding *Mycobacterium tuberculosis* (*Mtb*) dynamics within the host plays an important role. Previous studies on chronic active disease reported that *Mtb* accumulated genomic mutations within the host, possibly resulting in acquired drug resistance and increased virulence. However, several reports suggest that fewer mutations accumulate during LTBI than during the active disease, but the associated risk is largely unknown. Here, we analyzed the genomic dynamics of *Mtb* within the host during LTBI. Our results statistically suggest that *Mtb* accumulates mutations during LTBI, but most mutations are under low selective pressures, which induce mutations responsible for drug resistance and virulence. Thus, we propose that LTBI acts as a source for new TB disease rather than as a period for in-host genome evolution.

## INTRODUCTION

Tuberculosis (TB) remains the second leading cause of death from a single infectious agent; one-quarter of the world’s population is estimated to be infected with *Mycobacterium tuberculosis* (*Mtb*) (1, 2). Most people infected with *Mtb* are in a state called latent TB infection (LTBI), which is asymptomatic and noninfectious, while viable *Mtb* remains within hosts and can multiply to develop active disease (3, 4). Approximately 10% of LTBIs are estimated to develop into active TB disease in an individual’s lifetime (5). In countries with low TB incidence, most new active TB cases are predicted to be reactivations of LTBI (6, 7). Thus, preventing LTBI activation has become a significant strategy for TB elimination in these countries.

Understanding *Mtb* dynamics during LTBI is the key to controlling LTBI activation. However, in-host *Mtb* dynamics is more frequently analyzed in chronic active TB disease than in LTBI. Several studies of chronic active disease reported that *Mtb* and other Mycobacteria proportionately accumulate single nucleotide polymorphisms (SNPs) with disease duration (8–10). Typically, selective pressures, such as antibiotic treatments and host immune responses, increase mutations in specific genome positions responsible for the resistance to pressures and lead to the high ratio of nonsynonymous to synonymous mutation. Mutations detected in active disease show these characteristics, indicating that some mutations are under selective pressures (8–12). Furthermore, the *Mtb* population during active disease becomes genetically diverse, increasing their chances to adapt to environmental changes and survive within the host (13, 14). In addition to the scenario under active disease, previous studies also demonstrated the accumulation of SNPs and other genomic mutations in the *Mtb* genome during LTBI. Interestingly, while similar SNP accumulation rates were observed in macaques for LTBI and active disease, the former had a slower rate than the latter in humans (7, 15–18). However, whether other characteristics of genomic mutations, including population diversification and signs of selective pressures, are unique to LTBI or similar to active disease remains unknown.

We analyzed the genomic characteristics of *Mtb* from seven Japanese TB patient pairs with a strong suspicion of LTBI combined with public data. Using genetic homogeneity, SNP accumulation rate, and the proportion of nonsynonymous SNPs, changes in in-host genome evolution were compared and assessed between the *Mtb* population from LTBI and active disease.

## RESULTS

### Collection of genomes of the *Mtb* populations from LTBI and active disease

**Fig 1:**
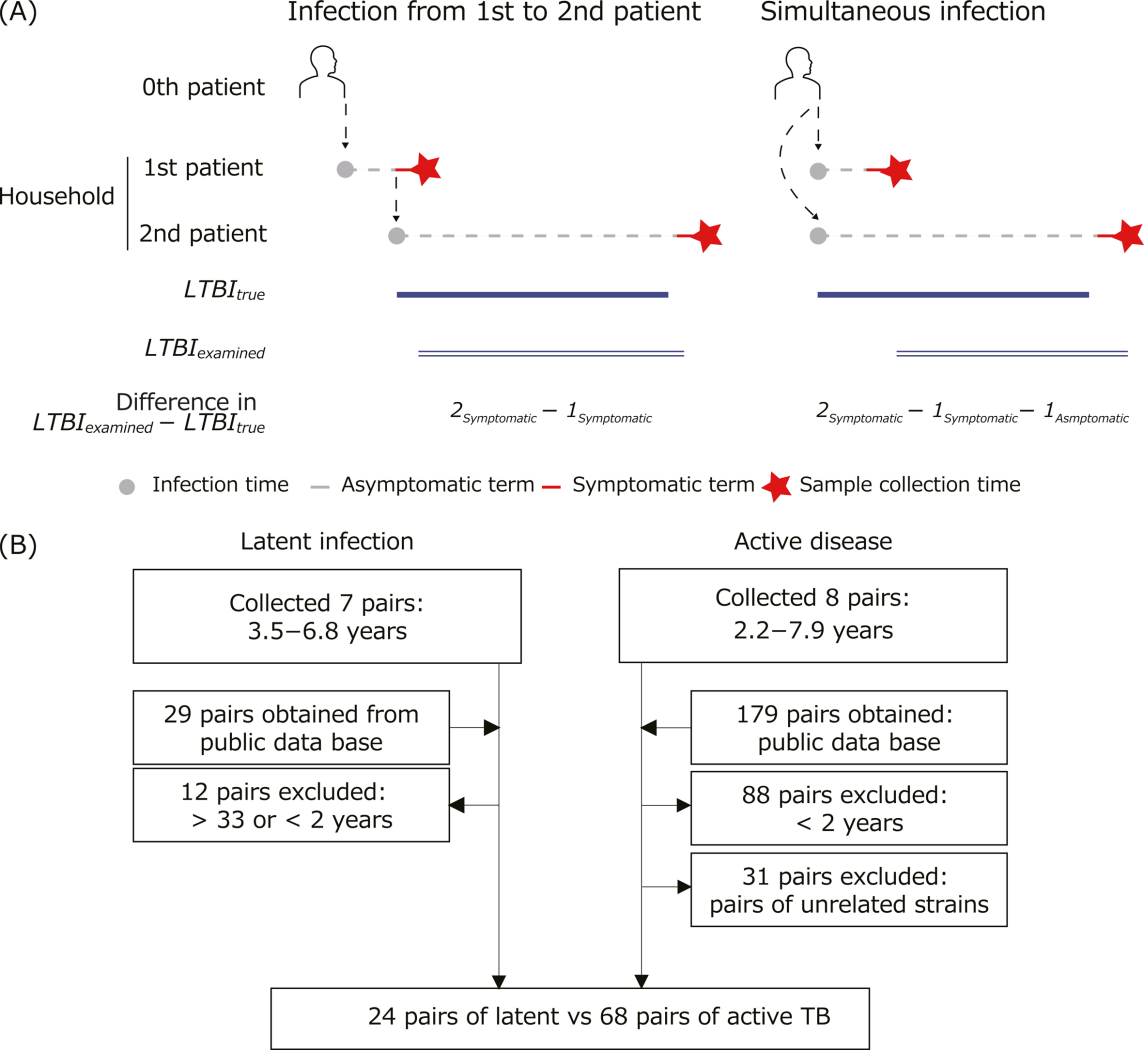
Analyzed *Mtb* samples with high suspicion of LTBI. (**A**) Schematic diagram of *Mtb* samples from patient pairs with strong LTBI suspicion and their time consideration in this study. Gray circles and red stars represent the dates of infection and the sample collection, respectively. Gray dotted lines represent the asymptomatic term after the infection and were described as *1_Asymptomatic_* and *2_Asymptomatic_* for first and second patients. Red lines represent symptomatic terms after developing active disease and were described as *1_Symptomatic_* and *2_Symptomatic_* for first and second patients. With high LTBI suspicion, *2_Asymptomatic_* sustained, and this period is regarded as LTBI period (*LTBI_true_*), described as blue lines, but could not be measured. Instead, differences in the sample collection dates, shown as the blue double lines, were used to infer the LTBI period (*LTBI_examined_*). If transmission occurs within a patient pair, *LTBI_true_* might differ from *LTBI_examined_* by *2_Symptomatic_ − 1_Symptomatic_* (left panel). If transmission occurs simultaneously to patients in a pair, *LTBI_true_* might differ from *LTBI_examined_* by *2_Symptomatic_ − 1_Symptomatic_ − 1_Asymptomatic_* (right panel). (**B**) Genome data sets analyzed in this study. *Mtb* genome data collected once from each of seven patient pairs with high LTBI suspicion and twice longitudinally from eight active disease patients, respectively, were obtained. These data were combined with publicly available *Mtb* genome data collected by equivalent method and filtered by the collection date differences within pairs and used for the analysis.

To characterize the *Mtb* genome during LTBI, we collected *Mtb* isolates from seven pairs of TB patients in Japan. Each pair consisted of two active TB patients, whose starting date of active disease differed by >3 years. The transmission between the two or simultaneous infection from another patient was implied since both patients lived in the same household and their isolates were genetically related (Table 1; Fig. 1A). Patients developing active TB earlier and later in each pair were defined as first and second patients, and *Mtb* isolates from first and second patients were named first and second samples, respectively. In these pairs, the second patient was suspected of having LTBI that reactivated to cause TB disease (Fig. 1A). Two out of the seven second patients had records of receiving drug treatment to prevent LTBI activation; this information was unavailable for others. The genomes of 14 isolates from the seven pairs were analyzed by PacBio HiFi long-read (hereafter referred to as HiFi) and Illumina short-read DNA sequencing. The assembled genomes from HiFi reads included genome sizes and gene numbers close to the reference strain H37Rv, suggesting complete genome constructions (Tables S1 and S2) (19). HiFi and Illumina reads were aligned to the assembled genomes, and their abilities to detect SNPs were compared (Fig. S1). Most SNPs were shared between HiFi and Illumina data regardless of the repetitiveness levels of genomic regions (Fig. S1A). The only exception was an SNP in a mobile genetic element present in multiple copies in the *Mtb* genome, for which the Illumina reads were incorrectly aligned (Fig. S1B). The same SNPs were detected when Illumina reads were aligned to the assembled genomes and H37Rv (data not shown). Thus, we considered that the SNPs detected by aligned Illumina reads to H37Rv were of enough quality for further analysis. Then, we searched publicly available Illumina data of *Mtb* genomes collected like our seven sample pairs and obtained sequencing data from 29 additional patient pairs (Fig. 1B; Table S3) (15, 16, 18, 20). We selected 19 pairs whose dates of sample collection differ by ≥2 years since most patients develop active TB within 2 years after infection, and LTBI becomes a quiescent state after an asymptomatic state ≥2 years (18, 21). To effectively compare these pairs with our sample pairs, whose durations were 3.5–6.8 years, we excluded two more pairs; the in-pair differences in dates of developing active disease were >33 years apart (15). We added the 17 data pairs to our seven sample pairs, resulting in 24 LTBI pairs.

**TABLE 1:** Collection dates of *Mtb* for seven household patient pairs with high suspicion of LTBI infection^*a*^

Patient pair	Primary sample		Secondary sample		Duration (years)
	Name	Date of collection	Name	Date of collection	
1	JPN-OP-1-1	January 2010	JPN-OP-1-2	August 2015	5.6
2	JPN-OP-2-1	June 2010	JPN-OP-2-2*	August 2016	6.2
3	JPN-OP-3-1	June 2012	JPN-OP-3-2	April 2019	6.8
4	JPN-OP-4-1	March 2012	JPN-OP-4-2*	October 2018	6.6
5	JPN-OP-5-1	January 2012	JPN-OP-5-2	July 2017	5.5
6	JPN-OP-6-1	December 2008	JPN-OP-6-2	May 2012	3.5
7	JPN-OP-7-1	June 2010	JPN-OP-7-2	May 2014	3.9

^*a*^ The *Mtb* collection dates from seven TB patient pairs are shown. Duration represents differences between dates in each pair. The diagnosis was made 1–3 months before the collection. Asterisks indicate available records of drug treatment for the prevention of LTBI activation. Note that it is unknown whether the other secondary patients received preventative treatment.

To characterize the *Mtb* genomes of the 24 LTBI pairs, we compared them with sample pairs of chronic active disease; in each pair, two *Mtb* samples were longitudinally collected after a period of ≥2 years (Table 2). We collected eight *Mtb* isolate pairs, in which date differences were 2.2–7.9 years, and obtained Illumina reads from them (Fig. 1B). These eight patients included those that were both treated and untreated with antibiotics during the treatment period (Table 2). According to the LTBI pairs, we defined *Mtb* samples collected earlier and later as first and second samples, respectively. We also retrieved publicly available Illumina sequencing data of 91 sample pairs of chronic active disease and excluded 31 pairs consisting of genetically unrelated strains (Fig. 1B; Table S3) (22–26). Since the pairs whose collection date differences were <2 years were filtered, SNP accumulation rates between the group including and excluding these pairs were compared, but no significant differences were observed (Fig. S2). Therefore, the 60 data pairs of active diseases collected ≥2 years apart were combined with our eight sample pairs and used for further analysis (Fig. 1B).

**TABLE 2:** Collection dates of *Mtb* and treatment history of the eight patients with chronic infection^*a*^

Patient	Date of collection		Duration (years)	Drug treatment
	Primary sample	Secondary sample		
CHR1	December 2002	March 2008	5.3	Partial
CHR2	March 2002	May 2004	2.2	Yes
CHR3	October 2002	July 2010	7.7	Partial
CHR4	June 2003	February 2008	4.7	Partial
CHR5	January 2009	January 2015	6	No
CHR6	July 2009	June 2012	2.4	No
CHR7	April 2013	November 2015	2.6	No
CHR8	July 2008	July 2016	7.9	Partial

^*a*^ *Mtb* collection dates from the eight patients with chronic TB and drug treatment histories. The duration represents the differences between collection dates. Drug treatment during the interval is grouped into three categories: yes, no, and partial, which indicates continuous drug treatment, no drug treatment, and a combination of periods with and without drug treatment, respectively.

Each sample of LTBI pairs was collected from different patients, while that of active disease pairs was collected from the same patient. Thus, in LTBI pairs, individuals in the *Mtb* population of first patients can have minor mutations, which can be transmitted to second patients (Fig. S3A). In such cases, the mutations are not detected, or low allele frequencies (AFs) in the first sample are detected, but high AFs in the second sample are detected, leading to the wrong interpretation that the *Mtb* population accumulated the mutations within the second patients by in-host evolution. To evaluate this situation, two genomes from each pair were compared and AFs were calculated. AFs of SNPs in the first samples were compared between pairs if these were ≤0.2 in the first samples and ≥0.9 in the second samples (Fig. S3B and C). AFs of SNPs in the first sample were scattered with the peak at zero in LTBI, while most of these were zero in active disease. SNPs in each of the first LTBI samples tended to have similar AFs (Fig. S3D). These results implied that transmissions of minor SNPs occurred between two samples of several LTBI pairs. To examine in-host evolutions and remove transmissions, SNPs within each pair of LTBI and active disease were used for further analysis, if AFs in the first sample are ≤0.01.

### *Mtb* populations from LTBI are genetically more homogeneous than those from active disease

**Fig 2:**
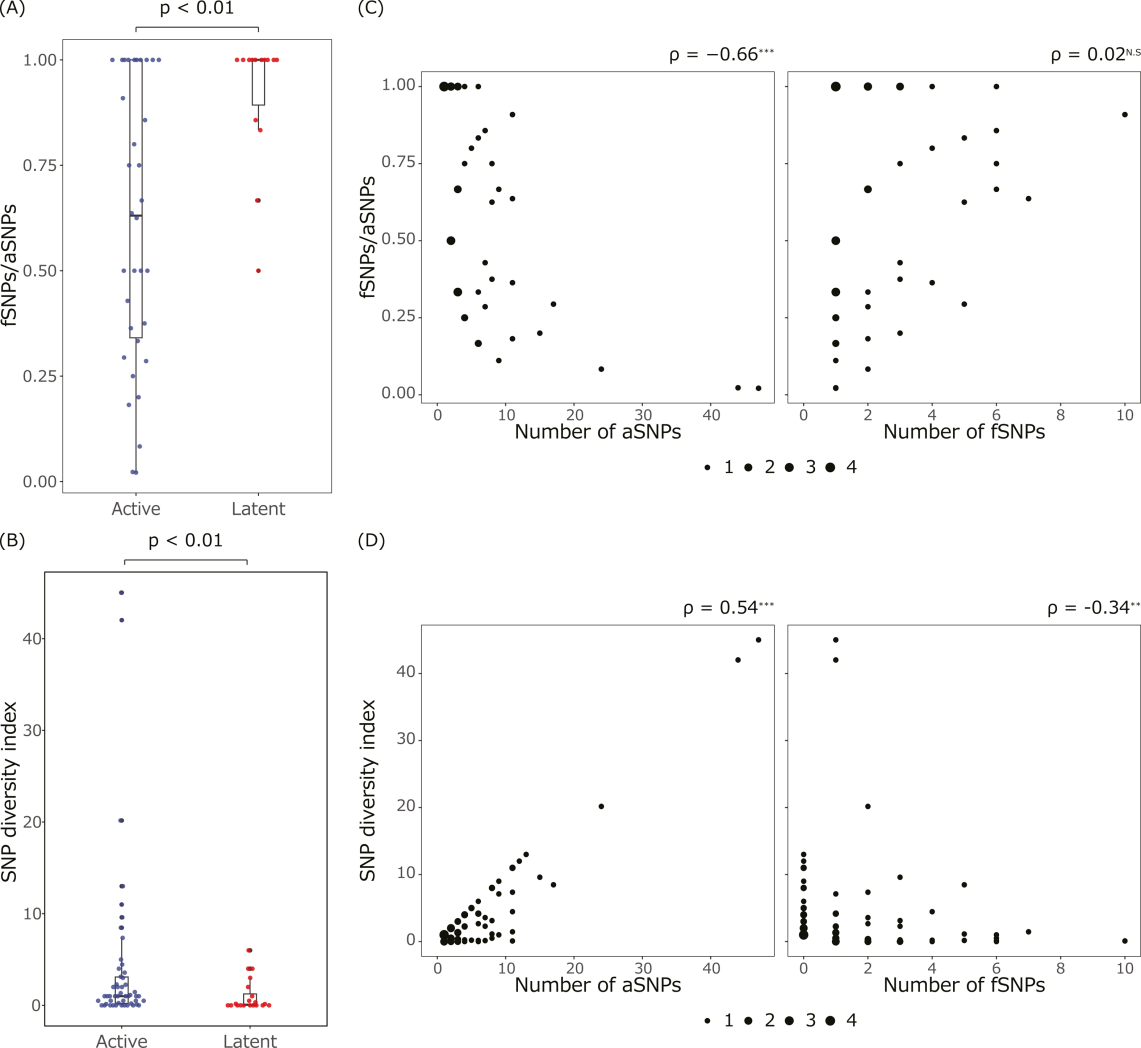
Homogeneity of the *Mtb* population in LTBI (**A and B**). (**A**) Ratio of fSNPs to aSNPs and (**B**) SNP diversity index in the *Mtb* population from active disease and LTBI. *P*-values of the Wilcoxon rank sum test between active disease and LTBI are shown at the top of the graph. Blue and red points represent data from active and LTBI, respectively. (**C and D**) Correlation of (**C**) the ratio of fSNPs to aSNPs and (**D**) the SNP diversity index with fSNPs and aSNPs in the *Mtb* population from active disease and LTBI. The size of the points represents the number of corresponding samples. The Spearman’s rank correlation coefficient is shown on the top right. Asterisks indicate significant correlation at *P*-values of <0.01 (**) and 0.001 (***), while N.S. indicates not significant. Samples from active disease (*n* = 34) and LTBI (*n* = 15), which produced ≥1 fSNP(s), were used for analysis.

To characterize the genetic diversity of *Mtb* populations during LTBI, the SNP ratios detected with high and low AF thresholds were compared with the active disease (Fig. 2A). The high and low AF thresholds were set at ≥0.9 and ≥0.2, respectively, and referred to detected SNPs as fixed SNPs (fSNPs) and all SNPs (aSNPs). aSNPs also include fSNPs, as shown in thresholds. AF ≥ 0.2 was chosen as an AF > 0.19 suffices for maintaining SNPs in the *Mtb* population, and all sequence data have enough depth to detect AF ≥ 0.2 (Table S4) (9). The *Mtb* population during LTBI had a higher proportion of fSNPs to aSNPs than the *Mtb* population during active disease (Fig. 2A). To assess the diversity, we further defined the SNP diversity index, an index to examine the ratio of fSNPs and aSNPs (see Materials and Methods), and these significantly differed in LTBI and active disease (Fig. 2B). Both the ratios and SNP diversity index correlated with the number of aSNPs but not with that of fSNPs (Fig. 2C and D). These results indicated that *Mtb* populations from LTBI had fewer aSNPs compared to fSNPs than those from active disease and were, therefore, genetically homogeneous.

The seven LTBI pairs and eight active disease pairs were treated according to the same processes, including sputum collection, culturing in media, DNA extraction, and sequencing. To examine the possibility of changes in the AFs in these processes, the AF distribution of the 15 samples was plotted (Fig. S4). All isolates from the active disease pairs had low AFs compared to those from the LTBI, as indicated in Fig. 2.

### *Mtb* genomes during LTBI accumulate aSNPs slower than those during active disease

**Fig 3:**
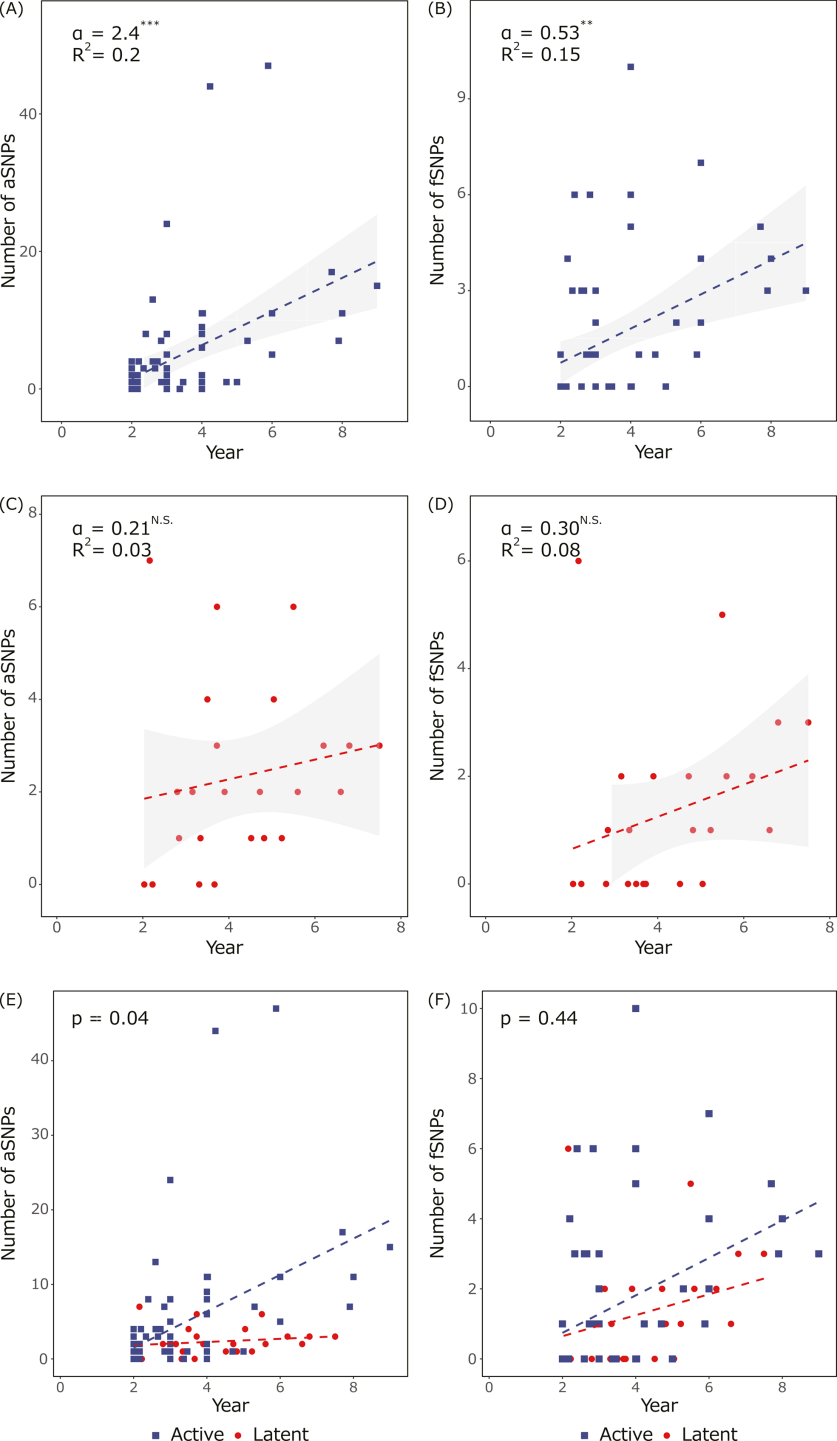
Comparison of SNP accumulation rates between the *Mtb* population from LTBI and active disease (**A–D**). Estimated accumulation rates of (**A and C**) aSNPs and (**B and D**) fSNPs in the *Mtb* population from (**A and B**) active disease or (**C and D**) LTBI. Dotted lines represent the estimated slopes. α and *R*^2^ indicate the estimated slope and Pearson’s correlation coefficient of linear regression, respectively. Asterisks indicate that coefficients are significantly different from zero at *P*-values of <0.01 (**) and 0.001 (***), while N.S. indicates no significant estimation. (**E and F**) Comparing the accumulation rate of (**E**) aSNPs and (**F**) fSNPs between the *Mtb* population from active disease (blue) and LTBI (red). Effects of the two infection types on the estimated slopes were tested by two-way ANOVA, and the *P*-values are represented on the top left. Dotted lines represent the estimated slopes with colors corresponding to points. Samples from active disease (*n* = 68) and LTBI (*n* = 24) were used for the analysis.

Due to fewer aSNPs in LTBI, we hypothesized that *Mtb* accumulates aSNPs slower than active disease. Therefore, SNP accumulation rates per year were estimated and compared between the *Mtb* population from LTBI and active disease (Fig. 3). Actual LTBI periods were unknown since the infection dates of the second patient were unclear (Fig. 1A). Instead, collection date differences of two samples in each pair were applied, which facilitated actual LTBI periods on the assumption that the duration of active disease before the sample collection (diagnostic delay) was similar between the two patients. In active disease, collection date differences were actual durations between two samples. A statistically significant linear regression of accumulation rates for aSNPs and fSNPs was found during active disease (Fig. 3A and B). Conversely, during LTBI, the SNP accumulation rates were not significantly estimated, as reported in a previous study (Fig. 3C and D) (18). Comparing the estimated slopes, it was observed that the *Mtb* population accumulated aSNPs slower during LTBI than during the active disease, while no difference was detected in fSNPs (Fig. 3E and F).

### Synonymous SNPs accumulated during LTBI correlate with latent periods

**Fig 4:**
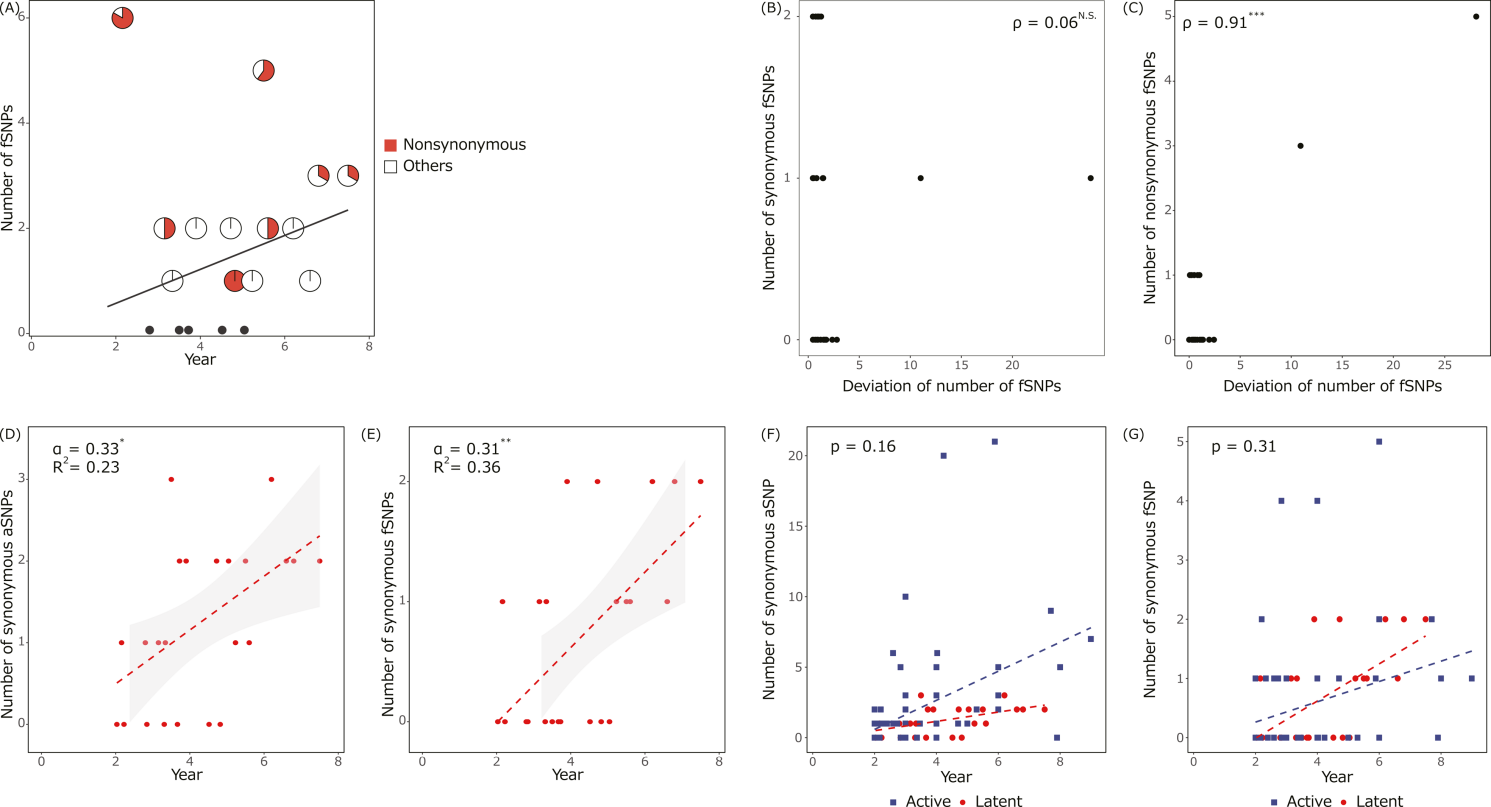
Estimated accumulation rates of synonymous SNPs in the *Mtb* population from LTBI. (**A**) Ratio of nonsynonymous (red) and other (white) fSNPs in the *Mtb* population from LTBI. The ratio is presented as pie charts and plotted with the estimated accumulation rate of fSNPs (black line). (**B and C**) Correlation between the deviation of observed fSNP numbers from the estimated fSNP numbers and observed number of (**B**) synonymous or (**C**) nonsynonymous fSNPs in the *Mtb* population from LTBI. The Spearman’s rank correlation coefficient is shown on the top right. Asterisks and N.S. indicate that the coefficient was significantly different from zero at a *P*-value of <0.001 (***) and not significantly different, respectively. (**D and E**) Estimated accumulation rates of synonymous (**D**) aSNPs and (**E**) fSNPs in the *Mtb* population from LTBI. Dotted lines represent the estimated slopes. α and *R*^2^ indicate the estimated slope and Pearson’s correlation coefficient of linear regression, respectively. Asterisks indicate that coefficients are significantly different from zero at *P*-values of <0.05 (*) and 0.01 (**). (**F and G**) Comparison of accumulation rate of synonymous (**E**) aSNPs and (**F**) fSNPs in the *Mtb* population from active disease (blue) and LTBI (red). Dotted lines represent the estimated slopes. *P*-values for the effects of two infection types on the estimated slopes by two-way ANOVA were represented. Samples from active disease (*n* = 68) and LTBI (*n* = 24) were used for the analysis.

Compared to the estimated line, several pairs of LTBI have more SNPs consisting of more nonsynonymous SNPs than others (Fig. 4A). Deviations of observed fSNP numbers from estimated fSNP numbers were correlated with nonsynonymous fSNPs but not with synonymous fSNPs (Fig. 4B and C). While accumulation rates of nonsynonymous SNPs fluctuate depending on the selective pressures, synonymous SNPs are relatively neutral; hence, only synonymous SNPs were proposed to be used to estimate a mutation clock (27). Then, the accumulation rate of synonymous SNPs was estimated in the *Mtb* population from LTBI and active disease (Fig. 4D through G). Coefficients in linear regressions of accumulation rates of synonymous SNPs significantly differed from zero in LTBI (Fig. 4D and E). No significant difference was seen in SNP accumulation rates for synonymous aSNPs and fSNPs between LTBI and active disease (Fig. 4F and G).

### *Mtb* populations during LTBI contain fewer nonsynonymous SNPs than those during active disease

**Fig 5:**
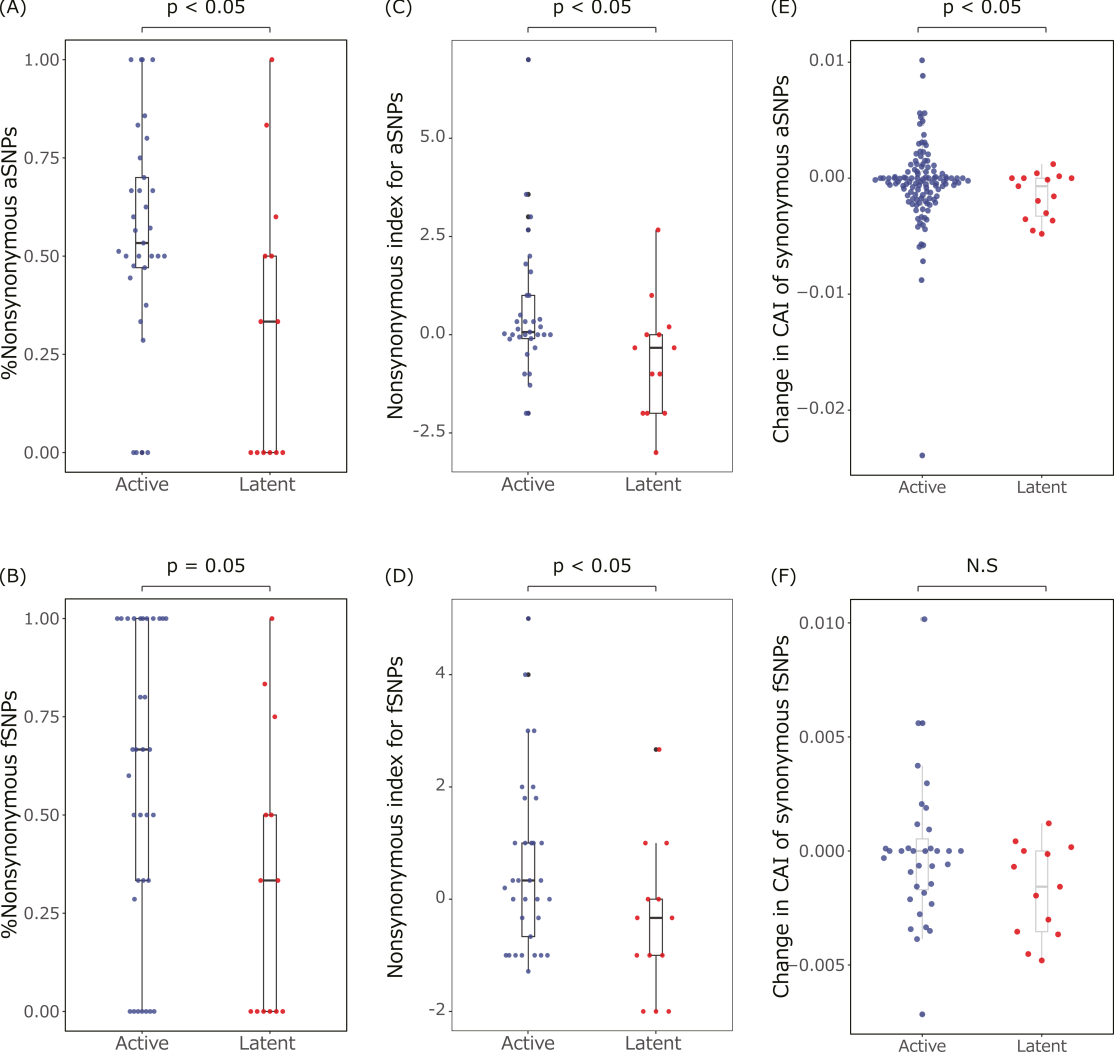
Proportion of nonsynonymous SNPs in the *Mtb* population from LTBI (**A–D**). (**A and B**) Ratio of nonsynonymous to genic SNPs and (**C and D**) nonsynonymous index of (**A and C**) aSNPs or (**B and D**) fSNPs in the *Mtb* population from active disease (blue) and LTBI (red). Samples from active disease (*n* = 34) and LTBI (*n* = 14), which produced ≥1 genic fSNP(s), were used for analysis. (**E and F**) Codon adaptation index (CAI) caused by synonymous (**E**) aSNPs or (**F**) fSNPs in the *Mtb* population from active disease (blue) and LTBI (red). The total numbers of synonymous aSNPs were 118 and 15 in samples from active disease (*n* = 29) and LTBI (*n* = 10), respectively. The total numbers of synonymous fSNPs were 35 and 13 in samples from active disease (*n* = 22) and LTBI (*n* = 10), respectively. *P*-values of the Wilcoxon rank sum test between active disease and LTBI were represented at the top of the graphs.

There are two selective pressures for genetically homogeneous populations (Fig. S5A and B). Under positive selections, selective pressures remove most of the population but keep individuals having mutations resistant to the pressures. Under purifying selections, emerging mutations are removed since most of the mutations are disadvantageous. To evaluate possible causes of homogeneity of the *Mtb* population from LTBI, selective pressures were assessed using nonsynonymous SNPs. Although the dN/dS is a widely used index calculated based on synonymous and nonsynonymous SNPs, it can be inefficient in this study since it is used to compare SNPs within independently evolved populations, and many sample pairs differ in only one or a few SNPs. Therefore, we instead used the percentage of nonsynonymous SNPs among genic SNPs and nonsynonymous index (see Materials and Methods) to assess the selective pressure (Fig. 5). *Mtb* populations from LTBI had fewer nonsynonymous SNPs than those from active disease for both aSNPs and fSNPs (Fig. 5A through D), indicating different selective pressures between them.

Patients with active disease should be treated with appropriate drugs according to guidelines (28); these treatments possibly induce and select nonsynonymous SNPs responsible for antibiotic resistance. Next, SNPs responsible for the resistance were compared according to the WHO mutation catalog (Table S5) (29). The number of SNPs matched to the reference mutation catalog and SNPs inside the genes found in the mutation catalog did not significantly differ between LTBI and active disease.

Recent studies indicated that synonymous SNPs contribute to fitness in the host environment; thus, synonymous, but not nonsynonymous, SNPs can be responsible for fitness during LTBI (30). Next, we examined the differences in the effects of synonymous SNPs on fitness using the codon adaptation index (CAI, Fig. 5E and F). CAI during LTBI decreased more than active disease regarding aSNPs, while these were not significant about fSNPs, indicating less contribution of synonymous aSNPs to fitness during LTBI.

### *Mtb* genomes during LTBI and active disease differ in the mutation spectrum

**Fig 6:**
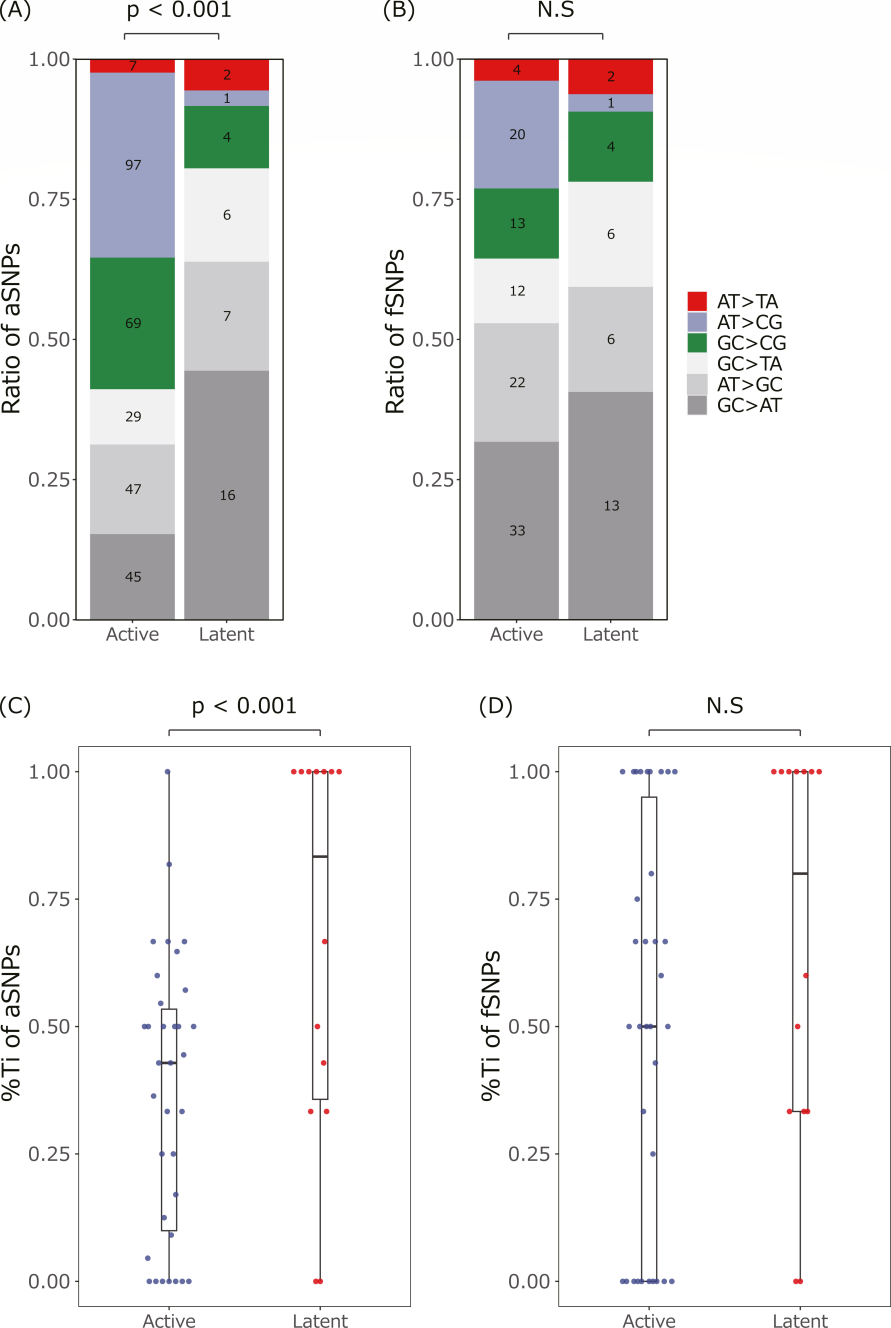
Mutation spectrum of SNPs in the *Mtb* population from LTBI (**A and B**). Mutation spectrum of (**A**) aSNPs and (**B**) fSNPs in the *Mtb* population from active disease and LTBI. *P*-values of the chi-square test are shown at the top of the graph, and N.S. represents non-significant differences (*P* > 0.05). (**C and D**) Transition (Ti) ratio of (**C**) aSNPs and (**D**) fSNPs in the *Mtb* population from active disease and LTBI. *P*-values of the Wilcoxon rank sum test between active disease (blue) and LTBI (red) were represented on top of graphs, and N.S. represents nonsignificant differences. *Mtb* populations from active disease (*n* = 68) and LTBI (*n* = 24) were used for the analysis.

Differences in the accumulation rate of aSNPs and the proportion of nonsynonymous SNPs raised the possibility that mutators differ between the *Mtb* population from LTBI and active disease. To examine this possibility, we compared the mutation spectrums affected by mutators’ identities (31) (Fig. 6). The mutation spectrum consisting of all detected SNPs significantly differed regarding aSNPs but not fSNPs (Fig. 6A and B). Some sample pairs have more SNPs than others, possibly skewing the spectrum if pairs did not weigh SNPs. To eliminate bias from spectrum comparisons, we compared the ratio of transition mutations (Ti) in each isolate (Fig. 6C and D). As before, the proportion of Ti differed significantly concerning aSNPs.

### *Mtb* genomes during LTBI accumulated structural variants and displayed an altered variable number of tandem repeats genotype

Structural variants between seven LTBI sample pairs were detected using the complete genomes assembled by HiFi reads (Table S6). Four structural variants were detected in two of the seven pairs. The relation of structural variant numbers to SNP numbers and infection duration could not be analyzed because of the small number of variants detected. Three of the four structural variants were found in loci used in variable number of tandem repeats (VNTR) analysis, which detects genetic relationships from the repeat sequence number across multiple loci (Table S6) (32, 33). Among the multiple loci, several are considered hypervariables that change more often than others. All three variants were located in loci classified as hypervariables, supporting the high occurrence of loci alterations. Another variant was found within the *pe-pgrs28* gene locus, which has repetitive sequences and, therefore, is considered prone to recombination. All structural variants detected from LTBI isolates occurred in recombination-prone regions.

## DISCUSSION

In bacteria, the frequency of mutations is positively correlated with the likelihood of acquiring drug resistance. Although *Mtb* also accumulates mutations during LTBI, the homogeneity (Fig. 2), slower mutation rate (Fig. 3 and 4), and lower proportion of nonsynonymous mutations (Fig. 5) suggested the existence of weaker selective pressures and a lower risk of acquiring mutations related to drug resistance and virulence during LTBI. Thus, we propose that LTBI acts as a source of new TB disease rather than as a period for in-host genome evolution.

There are three possible explanations regarding the *Mtb* population from LTBI pairs, which were more homogeneous than that from active disease but had a similar number of fSNPs. First, *Mtb* populations become diverse after the infection, and then selective pressures emerge and remove *Mtb* excluding individuals having adaptive mutations (Fig. S5C). Second, an individual in an initially small population accumulates mutations and grows with purifying selection (Fig. S5D). Third, individuals having mutations grow to occupy most of the population by random stochastic processes called genetic drift. We consider that the contribution of genetic drift is low since random stochastic processes should be similar between LTBI and active disease. We speculate that during LTBI, initial small *Mtb* populations accumulate mutations, as weak selective pressures are expected by a low nonsynonymous SNP ratio (Fig. 5). This correlates with the expected *Mtb* dynamics of TB activation after LTBI, wherein *Mtb* proliferates and accumulates mutations, but the host immune system sterilizes the *Mtb*, thereby keeping the population small. After LTBI activation, a few individuals within the small *Mtb* population proliferate, which includes the mutations accumulated during LTBI.

No significant difference was observed between fSNP accumulation rates in LTBI and active disease (Fig. 3F), but it might not be derived from the same mechanisms since the difference in selective pressures was indicated. Moreover, while a lower proliferation rate resulted in genetic homogeneity, different rates of aSNP accumulation between LTBI and active disease cannot be simply explained by the proliferation rate and should also be affected by selective pressures (Fig. 3E). One possible selective pressure is antibiotic treatment. Patients with chronic active disease should be treated with drugs according to the guidelines (28), while the 24 LTBI patient pairs in this study were a mix of untreated, treated to prevent LTBI activation, or no information about the treatments. Although the number of mutations responsible for the resistance during the period was not increased in active disease (Table S5), drug treatment could act as selective pressures to promote unknown resistance mutations and other nonsynonymous SNPs. This scenario can be assessed by comparing the *Mtb* population from LTBI with preventative drug treatment and active disease. In this study, two out of the seven second patients had received drug treatments to prevent LTBI activation after the first patients had developed active disease (Table 1), and for the others, the treatment histories were unknown. Although the two received drug treatment, neither had high mutation rates or high nonsynonymous mutations (data not shown), and additional samples are required to evaluate this. The existence of different selective pressures during LTBI and active disease was also indicated by the mutation spectrum (Fig. 6). GC > AT is putatively induced by oxidative stress and is the most common mutation in *Mtb* regardless of LTBI and active disease (9, 17). This was also observed in the study except for aSNPs during active disease, where AT > GC and CG > GC were increased (Fig. 6A and B).

One possible biological interpretation of the different AFs between the *Mtb* from LTBI and active disease may be determined from the lesion types (Fig. 2). A previous study reported that bacterial compositions differ between individual lesions within a host, regardless of LTBI or active disease (13, 14, 17). *Mtb* isolates collected from respiratory samples, such as sputum, are a mixture of bacilli produced from multiple lesions. They also reported a smaller number of lesions during LTBI (17). Another interpretation of our data is that the *Mtb* in each lesion acquires homogeneous mutations regardless of LTBI or active disease, and subsequently, bacilli from fewer lesions are expelled in the sputum of LTBI than active disease. The accumulation rates of all detected SNPs were not estimated by linear regression from LTBI (Fig. 3C and D). The results are consistent with previous reports in which the mutation rates could not be estimated, and different mutation rates between pairs less and more than 2 years apart were considered to be a major cause of the estimation fault (18). However, we could not estimate rates although the pairs whose collection dates differed ≥2 years were selected. Instead, synonymous SNPs could estimate the SNP accumulation rate from LTBI (Fig. 4D and E), as from active disease. Since synonymous SNPs are less affected by selective pressures than nonsynonymous mutations, they are more neutral and used to estimate molecular clocks (31). Indeed, samples with large deviations in the number of SNPs from the estimated line tend to have a high nonsynonymous SNP ratio (Fig. 4A through C), indicating nonsynonymous SNP fluctuated between samples, and thus, selective pressures differ between patients during LTBI. No significant difference was found in the estimated accumulation rates of synonymous SNPs between LTBI and active disease (Fig. 4F and G), while that of all aSNPs was slower in LTBI and active disease. It was reported that the mutation rate of all detected SNPs during LTBI is slower than that during active disease in humans, whereas it was equivalent in macaques (7, 15–18). Although our results from synonymous SNPs and all aSNPs comprised reports in macaques and humans, respectively, the sample size is statically small to reach any conclusion.

Regarding the limitations of this study, *Mtb* isolates were collected from sputum after developing active TB (Fig. 1A). Thus, isolates with high suspicion of LTBI might have experienced active disease and were not truly latent. To mitigate this problem, we applied a comparison between sample pairs; both samples in each pair experienced active disease, but the difference between them is expected to reflect what happened during the LTBI duration since the duration for developing disease is expected to be similar. We compared the difference in each pair between the *Mtb* population from LTBI and active disease, which enabled us to characterize mutations occurring during LTBI. However, it should be stressed that we reported genomic traits of LTBI compared with active disease, not absolute values; some mutations were accumulated during the terms of active disease after LTBI.

Another limitation of this study is that genomes were analyzed from the *Mtb* population grown in *in vitro* culture, where the composition of the *Mtb* population can alter. However, in active disease, most of the first samples were homogeneous, while the second samples were heterogeneous (Fig. 3B). Moreover, 15 sample pairs from LTBI and active disease grown under the same conditions represented the tendency of homogeneous and diverse populations, respectively (Fig. S4). These results exclude the possibility that the difference in homogeneity between the *Mtb* population from LTBI and active disease occurred in the culture process.

Finally, it must be emphasized that the seven sample pairs were “highly suspected of LTBI.” The two patients lived in the same household, their dates of developing active disease differed by more than 3 years, and their isolates differed by ≤5 fSNPs, thus evoking a high suspicion of LTBI (Table 1; Fig. 1). However, the possibility that they were independently infected with a related strain cannot be denied. In the latter case, our results should be interpreted quite differently.

Collecting and identifying *Mtb* isolates during or after LTBI are challenging because they require tracking for more than a few years after infection. In this study, seven patient pairs highly suspected of having LTBI were analyzed. Although persistent efforts to collect isolates and perform genotyping for >20 years enabled the collection of multiple sample pairs, a larger sample size would be preferable for statistical analysis. Molecular epidemiology using whole-genome sequencing has accelerated the detection of the genetic relatedness of isolates, and it will also accelerate the detection of numerous isolate pairs with high LTBI suspicion indicated by pairs with epidemiological contact, differences in developing dates of TB, and genetic relation by whole-genome sequencing. Isolates from sample pairs 2 and 5 were genetically related in whole-genome sequencing, but with a conventional genotyping method, VNTR, some loci diverged (Table S6). Similar TB cases where two isolates that were genetically related in whole-genome sequencing but differed in another genotyping method using mobile genetic elements were reported (7). Future large-scale analyses of *Mtb* isolates newly identified as LTBI by whole-genome sequencing could bring new insights.

## MATERIALS AND METHODS

### Growth conditions and DNA extraction of *Mtb*

Overall, 30 clinical isolates of *Mtb* were collected from sputum in Japan, out of which 14 were classified into seven pairs containing two isolates from a given household. The isolates in each pair were collected >3 years apart (Table 1). Since each pair had <12 SNPs, they were genetically related and were regarded as most likely LTBI infections. The remaining 16 isolates were grouped into eight pairs containing two isolates collected from the same patient with chronic active disease at intervals of ≥2 years. The *Mtb* isolates were cultured on 1% Ogawa medium, and grown isolates were stored at −30°C or −80°C. *Mtb* stocks were inoculated into Middlebrook 7H10 agar (Difco, Becton-Dickinson and Co., USA) supplemented with 10% Middlebrook OADC (oleic acid, albumin, dextrose, and catalase; Difco, Becton-Dickinson and Co., USA) and 0.5% (vol/vol) glycerol (Fujifilm Wako Pure Chem Co., Japan) and incubated at 37°C for 2 weeks. DNA was extracted from bacteria clumps in one volume of inoculation loops (4 mm) using the conventional phenol–chloroform method after bead beating (0.2 mm glass beads; Vortex Mixer GENIE2 with Microtube Attachment at max speed for 3 and 7 min for HiFi and Illumina sequencing analysis, respectively) (34). The quality and molecular weight of the genomic DNA samples were assessed by pulsed-field gel electrophoresis if these were analyzed by HiFi DNA sequencing.

### Whole-genome sequencing

To obtain HiFi reads, high molecular weight DNA samples >50 kb were sent to Macrogen Japan Co., Japan. HiFi SMRTbell libraries were prepared, and PacBio CCS subreads were obtained on the PacBio Sequel II System under a complete long reads mode (Pacific Biosciences of California Inc., USA) according to the standard protocol. To obtain Illumina reads, in our laboratory, TruSeq libraries were prepared and sequenced on the MiSeq II System (Illumina Inc., USA) with 300 bp paired ends according to the standard protocol.

### *De novo* genome assembly and variant calling from HiFi reads

The raw PacBio CCS subreads were converted into HiFi reads by ccs command v6.4.0 from the pbccs package of pbbioconda (Pacific Biosciences of California, Inc., USA) with default parameters. HiFi reads were assembled by flye v2.8.3 (35) to obtain the genomes. Genes in the assembled genomes were predicted by prokka v1.14.6 and compared by panaroo v1.2.8 (36, 37).

HiFi reads were aligned to the assembled genomes by minimap2 v2.24 (38). SNPs and structural variants were called by Deepvariant v1.1.0 and Sniffles v1.0.12, respectively, and filtered by the column FILTER “PASS” (39, 40). Repetitive regions were defined as those whose annotation by prokka included “PE family,” “IS,” “phage,” or “transposase.”

### SNP calling from Illumina reads

Sequencing data from *Mtb* sample pairs collected as our 15 sample pairs were obtained from NCBI Sequence Read Archive (SRA) (Table S3). Since the SNP accumulation rate was previously estimated as 0.56 SNPs/year (9), sample pairs with the number of fSNPs ≤ 0.56 × 5 and aSNPs ≤ 0.56 ×25 multiplied by duration years were regarded as the same strain-derived pairs. Sample pairs obtained from SRA are listed in Table S3 (*n* = 29 and 179 for LTBI and active disease, respectively), and the same strain-derived pairs of collection date difference ≥2 years were analyzed (*n* = 17 and 60 for LTBI and active disease, respectively). The Illumina reads from our samples and public data were aligned to the reference H37Rv genome (GenBank accession number: GCA_000195955.2) by BWA mem v0.7.17 (41). SNPs were called for each pair using bcftools mpileup tools and filtered by mapping quality >30 and depths of high-quality bases >20 in the FORMAT column and QUAL column >25 (42). AFs against H37Rv were calculated from allelic read depths of high-quality bases and converted into AFs against the major allele of first sample; if AFs against H37Rv were 0.80 in the first sample and 0.15 in the second sample, they were replaced by 0.20 and 0.85, respectively. aSNPs and fSNPs were defined as SNPs with AFs ≤ 0.01 in first sample and AFs ≥ 0.2 and ≥0.9 in second samples, respectively.

### Indexes to specify characteristics of SNPs

To assess population diversity, two indexes from fSNPs and aSNPs were calculated for each pair whose number of fSNPs ≥ 1. First, the ratio of fSNPs to aSNPs was calculated by $\frac{\sum fSNPs}{\sum aSNPs}$. It ranged from 0 to 1, decreased when the population was diverse, and was not affected by the number of SNPs. Second, the SNP diversity index was calculated by $\frac{{\left(\sum aSNPs-\sum fSNPs\right)}^{2}}{\sum aSNPs}$. It ranged >0, increased when the population was diverse, and increased when the total number of SNPs increased.

To assess the ratio of nonsynonymous SNPs, two indexes from synonymous and nonsynonymous SNPs were also calculated for each pair whose number of genic fSNPs ≥ 1. The ratio of nonsynonymous to genic SNPs was calculated by $\frac{\sum nonsynonymous\;SNPs}{\sum nonsynonymous\;SNPs+\;\sum synonymous\;SNPs}$. It ranged from 0 to 1, increased when the ratio of nonsynonymous SNPs increased, and was not affected by the number of SNPs. The nonsynonymous index was calculated by $\frac{\sum nonsynonymous\;SNPs-\;\sum synonymous\;SNPs}{\left|\sum nonsynonymous\;SNPs-\;\sum synonymous\;SNPs\right|}\times \frac{{\left(\sum nonsynonymous\;SNPs-\;\sum synonymous\;SNPs\right)}^{2}}{\left(\sum nonsynonymous\;SNPs+\;\sum synonymous\;SNPs\right)}$. It ranged from negative to positive and increased when the ratio and number of nonsynonymous SNPs increased.

The CAI was calculated for each synonymous SNP using the corresponding gene as a reference using Python v3.8.3 with CAI package v1.0.3 (43). For the collected LTBI samples, the assembled genomes were used as a reference for the analysis.

### Visualization and statistical analysis

All graphs were visualized using R v4.1.2 with ggplot2 packages. Wilcoxon rank sum tests, Spearman’s rank correlation tests, and chi-square tests were also conducted in R v4.1.2. SNP accumulation rates were estimated by linear regression using the lm function in R, and the difference of estimated slopes was tested by two-way ANOVA using the ANOVA function of the car package.

## Data Availability

Sequence data supporting the findings of this study have been deposited in the NCBI SRA database with accession numbers PRJNA1019874 and PRJNA1087460.

## SUPPLEMENTAL MATERIAL

Supplemental figures spectrum.04289-23-s0001.docx Fig. S1 to S5.

Supplemental material spectrum.04289-23-s0002.xlsx Tables S1 to S6.

ASM does not own the copyrights to Supplemental Material that may be linked to, or accessed through, an article. The authors have granted ASM a non-exclusive, world-wide license to publish the Supplemental Material files. Please contact the corresponding author directly for reuse.
