# Supplementary material for: Mycobacterium tuberculosis is less likely to acquire pathogenic mutations during latent infection than during active disease
Source: Microbiol Spectr. 2024 May 24;12(7):e04289-23. doi: 10.1128/spectrum.04289-23 (PMC11218478; doi:10.1128/spectrum.04289-23)
Supplement: Supplemental figures — Fig. S1 to S5. [file spectrum.04289-23-s0001.docx]

**Supplementary Information**

Table S1. The size of assembled genomes in the *Mtb* population analyzed in this study

Table S2. Number of predicted genes in the *Mtb* population analyzed in this study

Table S3. Sequence sample list obtained from NCBI Sequence Read Archive

Table S4. Average coverages of Illumina reads analyzed in this study

Table S5. Detected SNPs related to antimicrobial resistance

Table S6. Detected VNTR from LTBI pairs

Fig S1. Comparison of SNPs detected by HiFi and Illumina reads

Fig S2. Comparison of SNPs accumulated during active disease between different periods

Fig S3. Possible transmission of minor *Mtb* population within patient pairs with LTBI

Fig S4. Distribution of SNPs detected in the Mtb population from LTBI and active TB infection

Fig S5. Potential causes for a population with genetically homogenous mutations

| A | 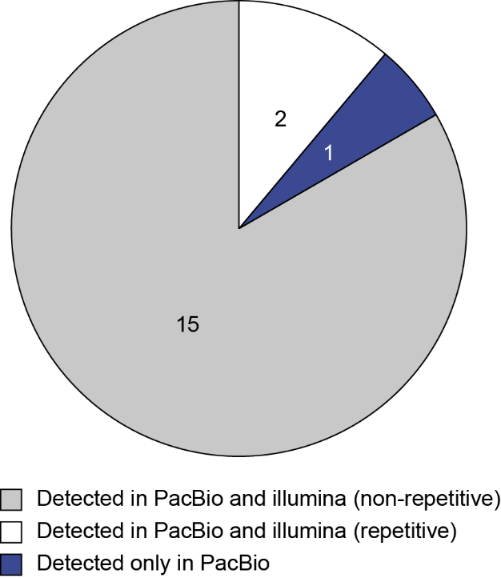 |
| --- | --- |
| B | 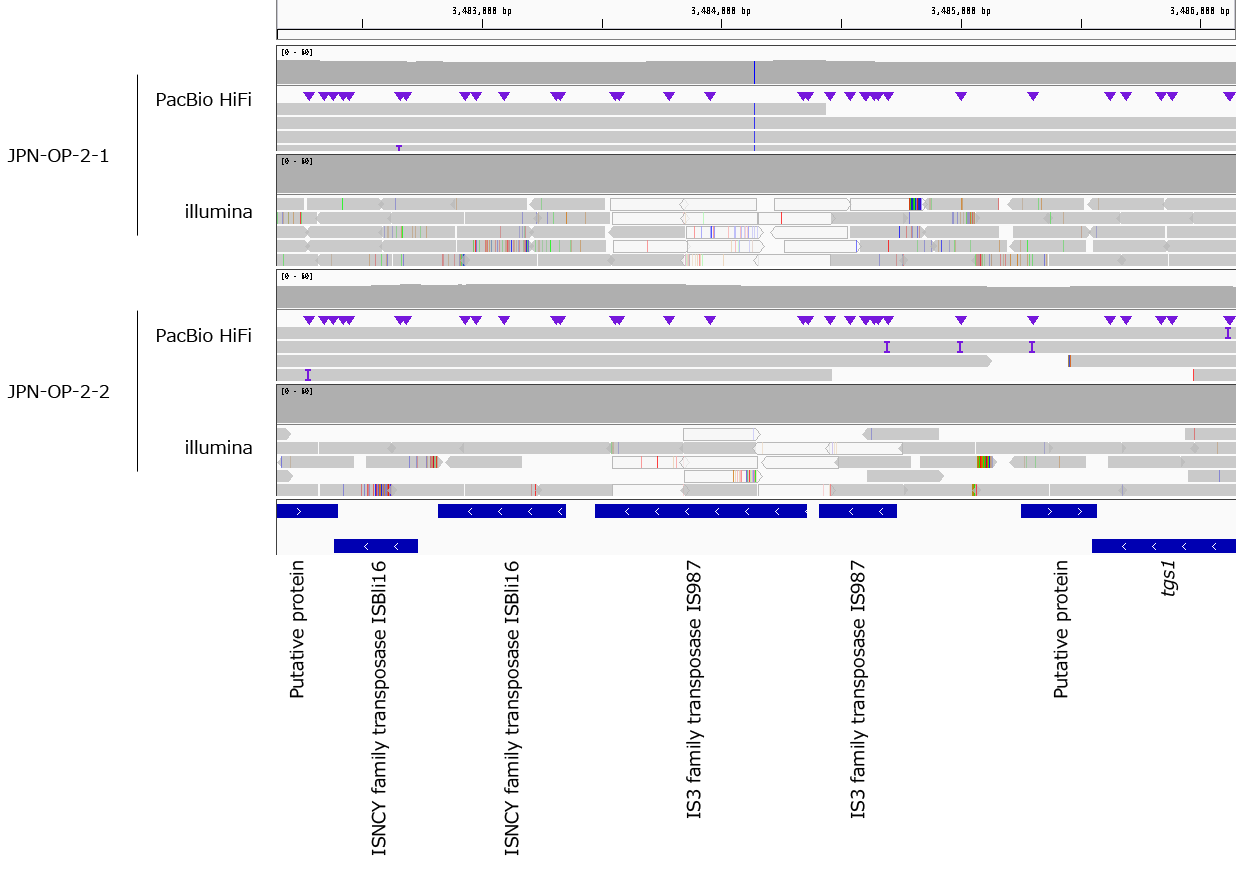 |

**Supplementary Figure 1. Comparison of SNPs detected by HiFi and Illumina reads**

(A) SNPs detected by HiFi and/or Illumina reads in *Mtb* from seven patient pairs with LTBI. SNPs were grouped by their location on the repetitive region in the *Mtb* genome (repetitive or nonrepetitive).

(B) Visualization of aligned HiFi and Illumina reads using Integrated Genome Browser (IGV). Each pentagon represents a read; the white pentagons are reads not precisely aligned to the reference genome. Predicted annotations are described at the bottom.

| A | 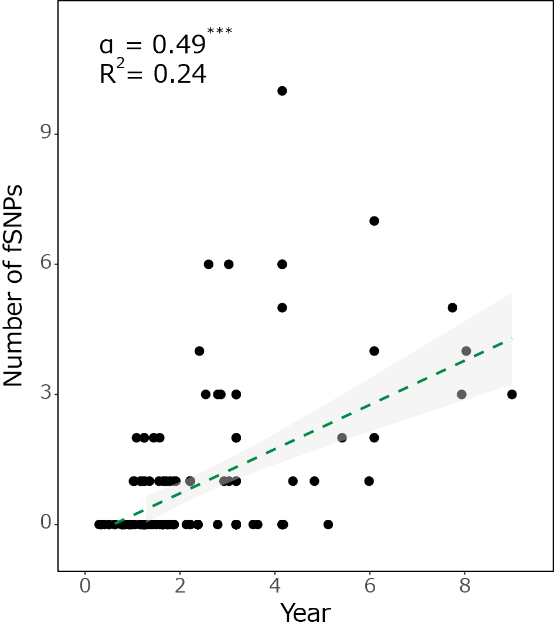 | B | 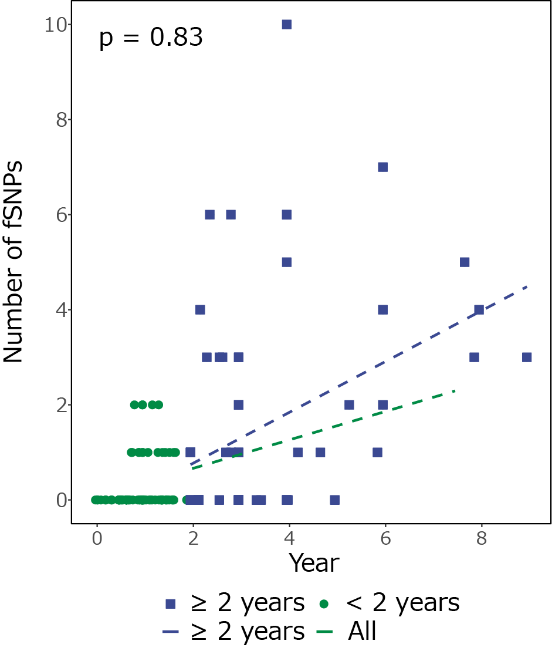 |
| --- | --- | --- | --- |

**Supplementary Figure 2. Comparison of accumulation rates of fSNPs during active** **TB disease between different periods**

(A) Estimated accumulation rates of fSNPs in the *Mtb* population from active TB disease. The dotted lines show estimated slopes. α and R^2^ indicate the estimated slope and Pearson’s correlation coefficient of linear regression, respectively. Asterisks indicate that coefficients are significantly different from zero at p-value of <0.001 (***).

(B) Comparison of accumulation rates of fixed fSNPs in the *Mtb* population from active disease between different periods. All periods (n = 136, green) and those of **≥**2 years (n = 68, blue) were compared. The effect of period difference on the estimated slopes was tested by two-way ANOVA, and its p-value is described on the top left.

| A | 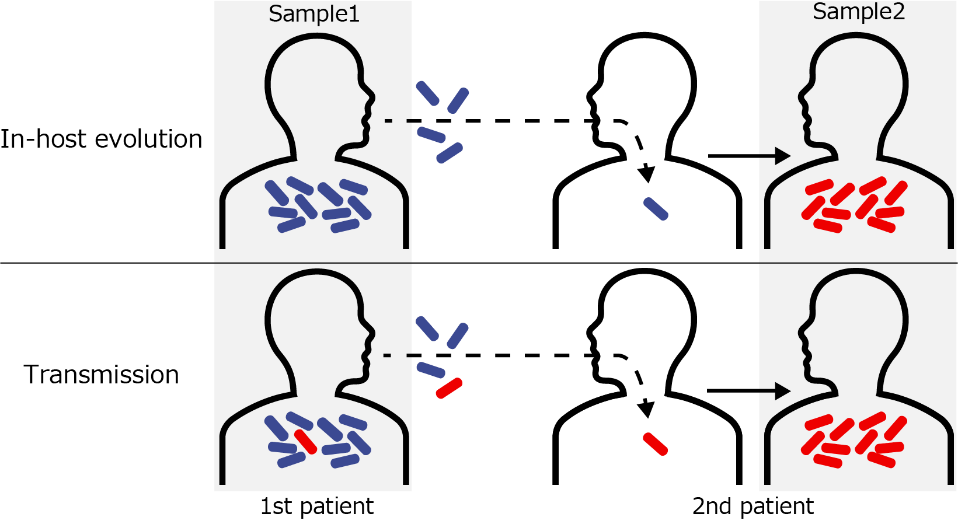 | | |
| --- | --- | --- | --- |
| B | 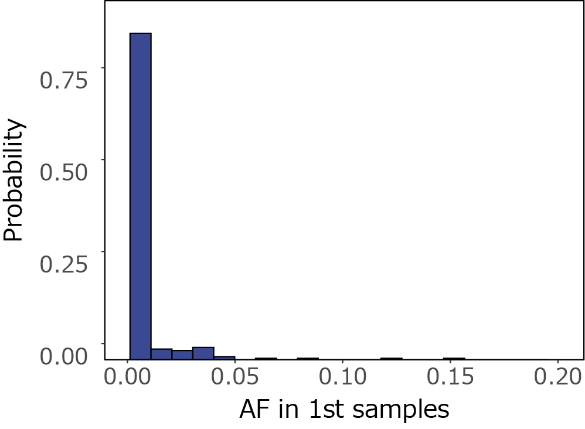 | C | 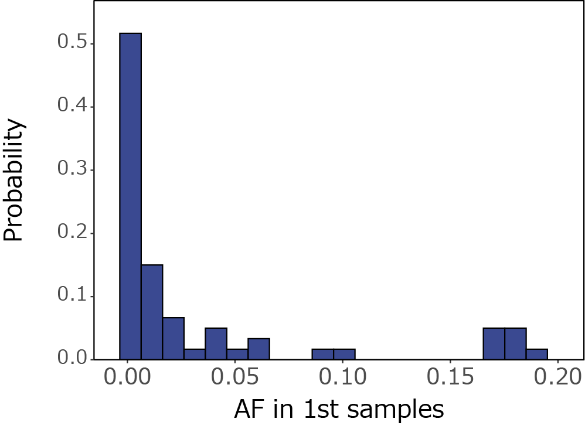 |
| D | 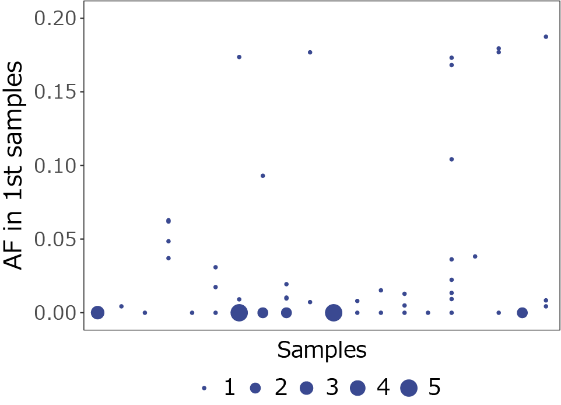 | | |

**Supplementary Figure 3. Possible transmission of minor *Mtb* population within patient pairs with LTBI suspicion**

(A) Two possible mechanisms showing fSNPs are detected in the *Mtb* population from a 2nd patient of LTBI pairs. In the upper case, *Mtb* accumulates SNPs inside the 2nd patient, and took the majority within the host. In the lower case, minor *Mtb* population in the 1st patient transmitted to the 2nd patient. The upper case is in-host evolution while the lower case is transmission. Dotted and straight arrows represent transmission and passage of time inside 2nd patients, respectively.

(B, C) Histogram of SNP AFs in the *Mtb* population from 1st patients in (B) active disease and (C) LTBI.

(D) Bubble chart of AF in the *Mtb* population from 1st patients of patient pairs of high LTBI suspicion. Sizes of points represent the numbers of SNPs corresponding to each AF. The X-axis represents different patients.

(B, C, D) SNPs were plotted on the graph when AF ≤0.2 and AF ≥0.9 in the *Mtb* population from 1st and 2nd patients, respectively.

*Mtb* populations from active disease (n = 8) and LTBI (n = 7) were used for the analysis.

| 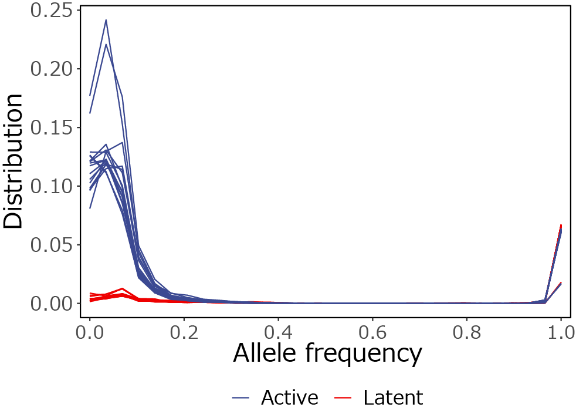 |
| --- |

**Supplementary Figure 4. Distribution of SNPs detected in the *Mtb* population from LTBI and active TB infection**

Distribution of SNPs that were detected in the *Mtb* population from active disease and LTBI. The SNPs were selected if either of the pair had an AF >0.05, and then the AFs of both of the pair were added to the data. The data for each pair was plotted on the graph. The

*Mtb* populations from active disease (n = 8) and LTBI (n = 7) were used for the analysis.

| 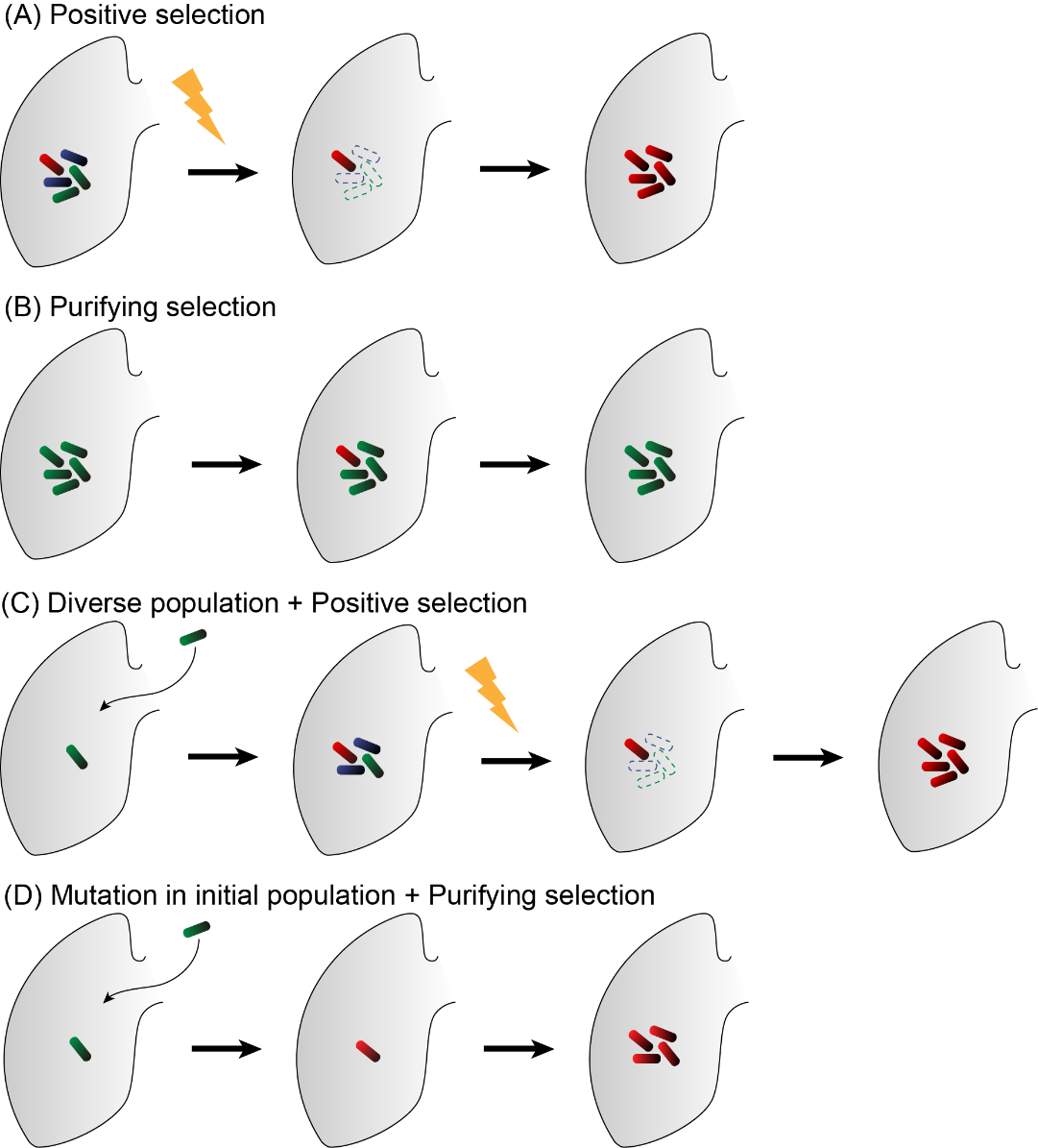 |
| --- |

**Supplementary Figure 5. Potential causes for a population with genetically homogenous mutations**

(A) Selective pressures remove the majority of the population except for individuals acquiring advantageous mutations to survive and the population becomes homogenous.

(B) Individuals in the population accumulate mutations, but hardly grow in the population, since the majority of the mutations are disadvantageous to survival. Thus, the population remains homogenous.

(C) After infections, individuals accumulate mutations, and the population becomes diverse without selective pressures. When selective pressures emerge, most of the populations are excluded leaving individuals with specific mutations, resulting in a homogenous population with fSNPs.

(D) Just after infections, most of the accumulated mutations except for the lethal ones are kept since there are few other competitors. After the population grows, purifying pressures remove emerging mutations, resulting in a homogenous population with initially acquired mutations.
